# Improved Leadership Skills and Aptitudes in an Excellence EMBA Program: Creating Synergies With Dialogic Leadership to Achieve Social Impact

**DOI:** 10.3389/fpsyg.2020.00017

**Published:** 2020-01-31

**Authors:** José Antonio Campos, Adriana Aubert, Mengna Guo, Mar Joanpere

**Affiliations:** ^1^Department of Marketing, University of Deusto, Bilbao, Spain; ^2^Department of Sociology, University of Barcelona, Barcelona, Spain; ^3^Department of Business Management, Universitat Rovira i Virgili, Reus, Spain

**Keywords:** social impact, business leadership, dialogic leadership, EMBA, organizational context

## Abstract

Psychological research on leadership has demonstrated that it achieves social impact, particularly in the improvement of working environments and organizational performance. The understanding of the organizational context of leader behavior and its different components is crucial to analyzing the impact of leadership in organizations. The purpose of this study is to identify and analyze the transformation and change of leadership skills and aptitudes before and after the implementation of an excellence EMBA program, particularly in relation to two components of the organizational context: (1) goals and purposes, and (2) people and groups. Data were collected from open-ended questionnaires completed by alumni and current participants in an Executive MBA program (EMBA) and enrolled in leadership courses. The emerging issues identified in the responses include themes linked to dialogic leadership and show that participants improve their leadership skills and aptitudes, advancing toward effective leadership and potential social impact in their organizations. The article concludes with a discussion identifying synergies with current developments of psychological research in leadership and the social impact of science.

## Introduction

Since the beginning of the 20th century, psychological research on leadership has contributed to generating improvements in business organizations that positively influence their performance and working environments, thus achieving social impact. Some of the most relevant scientific contributions analyze the effects of leadership on the improvement of interpersonal skills and the ability to reach personal and organizational objectives. In higher education studies, the research includes how to provide students with the necessary psychological competencies, skills, and abilities required to deal with a constantly evolving business climate (Thompson et al., 2019). Thus, a growing number of studies are focusing on the competencies required to generate an impact on business organizations, such as self-efficacy, leadership, and locus of control. In fact, leadership plays a key role in these studies in terms of the actions developed through leadership, their reception among organizational groups, and the impact generated on business organizations (Mumford et al., 2007; Thompson et al., 2019).

Among the contributions to classic psychological studies linked to leadership, Lewin et al. (1939) analyzed groups of children who experienced three leadership styles, demonstrating the effect of each one on the groups’ atmosphere and productivity. The initial psychological studies on the different types of leadership produced innovative contributions in the business context, showing how scientific research can generate social and economic improvements in business organizations. Moving forward to 2007, a special issue of “American Psychologist” introduced the latest theories and cutting-edge research on leadership, providing information that may motivate researchers to advance in leadership studies using a psychological perspective (Sternberg, 2007). The underlying questions in the special issue included aspects linked to the conditions in which leadership matters, the personal attributes of leaders that interact with situational properties to shape outcomes, and the reformulation of leadership models to treat all system members as both leaders and followers (Hackman and Wageman, 2007). The contribution by Bennis (2007) links leadership to the improvement of society and major world challenges. Furthermore, Avolio and Walumbwa (2006) advanced the knowledge related to building an integrative proposal of the developments and impact of psychological research to better understand leadership, paying special attention to cognitive elements and to the relationship between individual-follower behavior, among others. In this line, recent studies (Alase, 2017; Cai et al., 2018) analyze the inclusion of creativity into leadership. According to these contributions, the achievement of impact by leadership is related to motivating workers to develop objectives, enhance their perseverance, and generate different spaces to solve the same problem. Similarly, Chen (2007) suggests that teams are a key resource of new companies when these realize the potential for growth of the team’s creative ability and that business leaders can inspire employes to work together.

### Toward a Social Impact for Leadership and Psychological Research

Psychological research raises concerns about the well-being of citizens, including in the workplace, and how to develop research that has an impact on society. As in other disciplines, the impact of psychological research can occur in real or potential terms. Real impact implies that the research results have led to current improvements for society, whereas potential impact indicates some evidence of the effectiveness of research but that the results are not yet transferred (Pulido et al., 2018). Taking this into consideration, advancements in the field of leadership and psychology that address the decent work and economic growth of Goal 8 of the UN’s Sustainable Development Goals (United Nations, 2019) can contribute to real or potential impacts. Several authors and contributions have followed these approaches. House (2008) published a study focused on a historical analysis of social psychology, social sciences, and economics stating that economic power has grown exponentially during the last century thanks to the contributions of social sciences and psychology. Such developments are related to the promotion of inclusive and sustainable economic growth, employment, and decent work for all as assessed in the “Sustainable Development Goals Report 2019” (United Nations, 2019). Psychological research on leadership in organizations has had a relevant impact on management and economics studies. The 6th International Conference on Management, Leadership, and Governance (Riviere, 2018) focused on leadership knowledge in the context of three research dimensions: psychology, business economics, and project management. Some contributions highlighted the role of psychological training on leadership to achieve a social impact, particularly an economic impact. The impact of psychological research includes an improvement in the competencies and skills of undergraduate and MBA students, which also supports the advancement of international objectives focused on improving the economy and decent work.

### Approaching Leadership and Dialogic Leadership

Various fields of research have approached the study of leadership. On the one hand, studies in the fields of organizational behavior and industrial psychology focus on the negative features of leadership, such as abusive behaviors, toxic relationships, and bullying tactics (Harris and Jones, 2018). On the other hand, in the field of psychology, Vroom and Jago (2007) proposed that leadership is “a process of motivating people to work together collaboratively to accomplish great things” (p. 18). Chatman and Kennedy (2010) pointed out the need to study the theoretical approaches from psychology that allow leaders to develop skills and interact with small groups and large corporations. In this way, psychological studies in the field of business leadership can help inspire members of organizations to achieve organizational goals. According to Mumford et al. (2007), there is a need for in-depth psychological analysis to understand how leaders are able to influence others.

The conceptualization of diverse types of leadership has increased in recent years. Jardon and Martínez-Cobas (2019) developed a study based on the primary contributions of different types of leadership, such as ethical leadership, authentic leadership, spiritual leadership, and transformational leadership. Other authors (Bryant, 2003) relate the latter with transactional leadership, while Hitt and Duane (2002) study strategic leadership. Padrós and Flecha (2014) developed the conceptualization of dialogic leadership, defined as the process of creation, development, and consolidation of the leadership practices of diverse community members. The authors believe that one cannot reduce leadership to attributing a role to a person and not to the rest; rather, it has a human dimension. Leaders, in their dialogic responsibility, discover the required mechanisms for working together to support and promote actions that enhance changes in the organization and beyond. The conceptualization of dialogic leadership relates to entrepreneurial leadership, understood as a mechanism aimed at taking advantage of the creative potential of workers, supporting trust-building in the creative processes of organizations (Cai et al., 2018).

### Creativity and Heterogeneity: Toward Effective Leadership

In business, leaders drive or influence workers as role models (Más-Machuca, 2014; Renko, 2017). Some studies indicate the need for leadership to motivate workers with support and perseverance, facilitating the emergence of creative proposals from workers on their own (Gupta et al., 2004; Chen, 2007). In this sense, Cai et al. (2018) explain that entrepreneurial leaders should motivate team members to support benefits for both the team and its members. Furthermore, the authors state that the daily management of the company generates a specific type of leadership aimed at driving people toward creativity and new opportunities. On the basis of common objectives and a shared vision of the organization, the achievements of a group will generate a specific type of leadership. In this sense, leadership with a strong influence on business and able to thrive and work in a creative and innovative way supports successful business (Gupta et al., 2004).

Several studies approach the analysis of heterogeneity as comprising dimensions influencing effectiveness in organizations. Workforce diversity–defined as similarities and differences among employes in terms of age, cultural background, physical abilities and disabilities, race, religion, gender, and sexual orientation–is a key to improving productivity (Saxena, 2014) and makes the workforce heterogeneous. It is accompany the development of population mobility, fierce competition, and the expansion of the global market (Jackson et al., 2003). Scholars are trying to prove the relationship between heterogeneity and team performance through research. Hoch (2014), in examining diversity, sharing leadership, and information sharing, emphasized diversity as one of the conditions that influence the relationship between shared leadership and performance. In a similar vein, Kearney and Gebert (2009) highlighted the importance of transformational leadership, that is, when levels of transformational leadership are high, nationality and educational diversity are positively related to team leaders’ longitudinal ratings of team performance. Saxena (2014) emphasized the role of proper management, affirming that hiring a diversified workforce will definitely lead to improved productivity.

### Leadership Components of the Organizational Context

The scientific literature in the field of psychology includes analyses of very diverse components of leadership development in organizational contexts. Several studies have arrived at a possible agreement on the most relevant ones. According to Porter and McLaughlin (2006), the most relevant components identified in a review of leadership from 1990 to 2005 were culture/climate, goals/purposes, people/composition, processes, status/condition, structure, and time. Given that this study focuses on the leadership components of goals/purposes and people/composition, a more detailed description is provided.

Several scientific contributions introduce the relevant organizational context for the creation of leadership in relation to organizational goals and purposes. For instance, in the nursing field, a recent study identifies the essential components of nursing leadership as envisioning goals on the basis of what occurs in a specific context. In this respect, according to Miles and Scott (2019), “the context includes the follower’s commitment to leaders, socio-cultural realities, gender bias, situational realities, as well as the social, legal and political environment” (p. 9). A similar approach in tourism research notes that when leaders use empowering behaviors such as enhancing meaningful work or fostering autonomy, they develop supportive organizational structures that empower employes in a way that creates positive attitudes and promotes organizational goals (Amor et al., 2019).

The people/composition component is based on individual potential and its impact on leadership or performance (Porter and McLaughlin, 2006) and appears in the scientific literature in diverse forms. Miles and Scott (2019) studied the leadership curriculum framework to select skills capable of demonstrating a positive influence on others, identifying appropriate and inappropriate leadership and management behaviors, attitudes, and styles, or assessing personal strengths and weaknesses related to management and leadership in nursing, among other professions. The proposed skills show the relationship between individual and collective factors, which determines the group’s interactions and the possibilities of developing the proposed goals. In a similar vein, other studies (Salas-Vallina et al., 2018) advocate for leadership that enhances aspects such as happiness and well-being at work, focusing on interactions and reciprocal links.

### The Present Study and the Context of the EMBA

The purpose of this study is to identify and analyze the transformation of and changes in leadership skills and aptitudes before and after the implementation of an excellence EMBA program, specifically in relation to two components of the organizational context: (1) goals and purposes and (2) people and groups. The EMBA program at the core of this study is an advanced interdisciplinary course for senior managers who must hold responsible positions in companies. The primary purpose of the program is to improve the knowledge and abilities of the participants, making them directly confront professional challenges. The content of the EMBA program includes analyzing the influence of external factors on company performance, cultivating one’s leadership and decision-making capacity, exchanging rich experience among different managers in various industries, and supporting participants to create an interpersonal connection.

The program is divided into three horizons to respond to globalization challenges, leadership, and the integration of the various elements comprising a company. The first horizon pursues the idea that company executives must continue to understand the changing trends of the business environment and control the latest business operations rules. The second horizon is 21st-century company strategic thinking, as the traditional view based on pre-established patterns is no longer sufficient to respond to changes in business trends. The third horizon is a strategic vision centered on decision-makers. Training executives show them how to use leadership, motivation, and negotiation to guide others to follow their thinking. This particular horizon highlights research into personality, including how to solve difficulties through behavioral analysis and communication skills.

Developmental psychological research in leadership includes topics such as building individual capacities to lead change, improving a shared vision among people, and heterogeneity in working groups, among others. However, less is known about the development of these topics in excellence EMBA programs addressed to leaders in business organizations. This article focuses on the organizational components of goals/purposes and people/composition to analyze to what extent their development has an impact on the leadership skills and aptitudes of participants.

## Materials and Methods

### Study Design

We conducted this study using open-ended questionnaires filled out by alumni and current participants of an EMBA at the University of Deusto (Bilbao, Spain) whose syllabus offers several leadership courses. The open-ended questionnaire was sent via an email providing a link to obtain qualitative data on interpretations of, reflections on, and self-evaluation of the improvements resulting from the EMBA in the development of leadership skills and attitudes. This approach considers that the improvement in leadership skills and aptitudes may have a potential social impact on organizations.

### Participants

The EMBA involving the participants in this study targets professionals in managerial positions (such as commercial directors, technical and financial managers, etc.) in specific areas of business organizations, the idea being that they can assume new challenges and make the leap to top management. Participants in the EMBA come from different industries and sectors, resulting in a heterogeneous group. Including the 2018–2019 academic course, there have been ten offerings of the EMBA, producing approximately 250 alumni, and another is underway. All alumni and current participants were given information about the study and invited to participate.

The open-ended questionnaire was completed by 28 alumni and seven current participants in the EMBA. Of the 28 alumni, 69% were male and 31% female. Their ages ranged from 34 to 59 years old. Among them, six alumni were 44 years old, accounting for 21.43% of all alumni, followed by those 34 years old, accounting for 10.71% of past respondents. Five alumni were 35, 36, 38, 41, and 49, accounting for 7.14%. The number of 2019 graduates was the highest, five, accounting for 5% of all alumni, followed by students who graduated in 2013, 2015, and 2018, with four alumni of each year participating in the open-ended questionnaire in this study (Table 1).

**TABLE 1:** EMBA Alumni profiles (Alumni = A).

Questionnaire number	Age	Gender	Bachelor’s degree	EMBA graduation year	Responsibility in the company	Main activity of the company
A1	44	Female	BMA	1999	Senior manager/director	Strategic consulting
A2	47	Man	Business and marketing	2010–2011	Corporate and operations director	Insurance
A3	42	Man	Industrial organization engineering	2011	Manufacturing directorate	Operations
A4	36	Man	Industrial engineering	2011	Directorate general	Cold-stamping metals
A5	49	Man	Sociology; law	2013	Incubator manager	Education
A6	48	Female	Engineering	2013	Functional direction	Building
A7	38	Man	Telecommunications engineering	2013	Department manager	Railway signaling
A8	41	Man	Chemical sciences	2013	Direction	Transport paint marketer
A9	36	Man	Industrial electronics and automatic engineering	2014	General address	Manufacture and design of electronic boards
A10	41	Female	Engineering	2014	Functional address	Building
A11	50	Man	Engineering	2015	Managing director	Industrial
A12	44	Man	Engineering	2015	Head of area in project management	Rail transport systems
A13	44	Female	Doctor of informatics	2015	Entrepreneur and intermediate management	Telemedicine
A14	37	Female	Bachelor’s degree	2015	Freelance/entrepreneur	Business consulting
A15	59	Female	Telecommunication engineering	2016	General-partner address	Consultancy
A16	43	Man	Tourism	2016	CEO subsidiary United States	Data analytics
A17	39	Man	Telecommunications engineering	2017	Middle management	New business development (security + Big data)
A18	35	Female	Humanities: communication	2017	Middle management	Sanitary
A19	34	Female	Telecommunications engineering	2018	Middle management	Industrial equipment electrical sector
A20	44	Man	Law	2018	Directorate general	Public administration
A21	44	Female	Electronics engineering	2018	Department director	Train manufacturing
A22	38	Man	Engineering	2018	Partner	Consultancy
A23	35	Man	Industrial engineering	2019	Project manager	Manufacture of flexible packaging for all types of markets
A24	40	Man	Industrial engineering	2019	Department address	Rail services
A25	34	Man	Computer engineering	2019	Business unit director	Engineering
A26	49	Man	Industrial engineer	2019	Directorate general	Cold-rolled steel
A27	34	Man	Engineering	2019	Systems technician	Railway industry
A28	44	Man	Postgraduate	2019	Intermediate business	Banking

In the case of current EMBA participants, seven males were involved in this study, two of whom were 31 years old, two were 35, one was 36, one was 38, and one was 44. Overall, current EMBA participants range in age from 31 to 44 (Table 2).

**TABLE 2:** Profiles of participants currently involved in the EMBA (current participants = CP).

Questionnaire number	Age	Gender	Bachelor’s degree	Responsibility in the company	Main activity of the company
CP1	35	Man	Ph.D.	Directorate general	Biosanitary
CP1	31	Man	Industrial engineering	Functional direction	Automotive
CP3	31	Man	Industrial engineering	Functional direction	Development and production of consumer electronics
CP4	35	Man	Industrial engineering	Engineering coordination and project management	Turnkey energy projects
CP5	36	Man	Science and food technology	General address	Production, distribution, marketing, and sale of bakery products and pastry
CP6	44	Man	Industrial engineering	Head of engineering and general services	Management of investments, energy, works, large projects, and warehouses; member of the steering committee
CP7	38	Man	Bachelor’s degree	Intermediate control	Industrial

### Design of the Open-Ended Questionnaire

The design of the core sections of the open-ended questionnaire follows three stages: a literature review to identify relevant components, the definition of synergies with leadership components of the EMBA, and the incorporation of the dialogic dimension of leadership.

First, we reviewed which potential leadership components to include in the questionnaire according to the literature review. The selection was based on literature linked to leadership and psychology that is relevant in the organizational context of leadership behavior. According to Porter and McLaughlin (2006), there is no universally agreed-upon set of components or other types of behavior occurring within an organizational setting, but there is a fair degree of consensus about seven components: culture/climate, goals/purposes, people/composition, processes, status/condition, structure, and time.

Second, we defined the synergies between the components identified in stage one and the leadership contents of the EMBA courses “Organizational Behavior” and “High-performing teams.” “Organizational Behavior” provides knowledge of the fundamental aspects involved in leading people in organizations, including understanding human behavior and motivation in the workplace, self-leadership, obtaining trust and commitment, establishing authority, developing talent via coaching skills, and creating a sense of mission and purpose. We identified the components of goals/purposes and people/composition as being the most suited to this approach. The “High-performing teams” course involves analyzing the characteristics of successful leaders, situational leadership, and the most important aspects of the results that a leader must have. Here we also identified goals/purposes and people/composition as being the most suitable for the questionnaire.

Third, we included the dialogic dimension in the open-ended questions, considering, in particular, the components of goals/purposes and people/composition identified in stage one and the contents of the EMBA courses highlighted in stage two. In this way, we specifically focused on the skills and aptitudes required to facilitate leading a diverse group and to promote a shared vision that leads to improving organizations, an approach that puts people at the center of changes.

As a result of these stages, we defined two core sections in the open-ended questionnaire: the leadership, objectives, and mission of the organization and people-oriented leadership (Tables 3, 4). Furthermore, we incorporated two other sections: individual data and a section introducing the major issues of the EMBA.

**TABLE 3:** Open-ended questionnaire of the core section: leadership, objectives, and mission of the organization.

For alumni		For current students	
1.	Lead people and/or teams in your organization; have you reverted to generating and consolidating a shared vision of what the organization is and what its purpose is? Why?	1.	Lead people and/or teams in your organization; do you invest in generating and consolidating a shared vision of what the organization is and what its purpose is? Why?
2.	Do you think that creating a sense of mission and purpose in your organization comes down to the effectiveness of groups and people? Why?	2.	Do you think that creating a sense of mission and purpose in your organization comes down to the effectiveness of groups and people? Why?
3.	What knowledge of leadership do you consider most useful in consolidating the objectives of the organization in working groups?	3.	What knowledge of leadership do you think can be more useful in consolidating the objectives of the organization in working groups?
4.	After taking your master’s degree, have you changed your perception of how people in your organization understand its objectives? In what sense?		

**TABLE 4:** Open-ended questionnaire of the core section: people-oriented leadership.

For alumni		For current participants	
(1)	What improvements do you identify from what you learned in the EMBA in relation to the development of successful leadership?	(1)	Are you able to identify weaknesses and strengths in members of your team? In what sense and/or aspects?
(2)	Are you now more able to identify weaknesses and strengths in members of your team? In what sense and/or in what aspects?	(2)	To what extent do you think the heterogeneity of individuals favors greater effectiveness in the performance of your work?
(3)	To what extent do you think the heterogeneity of individuals favors greater effectiveness in the performance of your work?	(3)	Do you think that the context can be improved so that different people in your organization can develop leadership? Why?
(4)	Do you think that, after taking your master’s degree, you have been able to improve the context for various individuals in your organization to develop leadership? Why?		

### Contents of the Open-Ended Questionnaires

We formulated two questionnaires with four sections each. One open-ended questionnaire was addressed to current participants of the EMBA and another to the alumni that had finished their EMBA. The underlying sections and questions were coherent between the two questionnaires.

Open-ended questionnaire for current participants. The first section of the questionnaire collects personal data on the participant, including their age, gender, grade degree, responsibility in the organization, and principal activity. The purpose of the second section is to identify the participants’ background of leadership prior to the EMBA, addressing whether they had leadership training and what their existing knowledge of leadership roles was. The third section identifies the participants’ perspectives on leadership and the objectives and mission of the organization: investment in a shared vision, the relationship between effectiveness and creating a sense of mission and purpose, and the consolidation of organizational goals. Finally, the objective of the fourth section is to determine participants’ perceptions of people-oriented leadership by asking them to identifying team members’ weaknesses and strengths, their views of the relationship between the degree of membership heterogeneity and job performance, and their views of the effect of improving the context in developing people’s leadership skills.

Open-ended questionnaire for alumni. The first section includes personal information and adds a question about “Graduation Year.” The second section adds a question about the EMBA’s assessment of leadership knowledge. The goal of the third section is to understand participants’ perceptions of leadership and the objectives and mission of the organization and identifies changes in their perceptions of how people in the organization understand its objectives after completing the EMBA. The fourth section asks for participants’ views on people-oriented leadership. In addition, it includes a question about the improvement in participants’ people-oriented leadership capabilities after completing the EMBA.

### Data Collection

The EMBA-DBS Director emailed the current participants and alumni with an invitation to complete the open-ended questionnaire via a link with the questions. The responses were automatically collected and shared with the research team in order to identify themes and subthemes appearing in the core sections.

### Data Analysis

The analysis involved identifying preliminary categories across responses, with particular attention to the core sections, 2 and 3. The preliminary categories were refined with a second and third round to review all responses, implicating different team members and linking categories and subcategories until the final list of themes was agreed upon. The names of the categories and subcategories were defined according to their presence and relevance in the responses.

## Results

All categories and subcategories were established inductively after a thorough reading of all responses from alumni and current participants. The categories of the current participants are heterogeneity in teams, dialogue and communication, goals and consensus, and group membership. The categories and subcategories of alumni are heterogeneity in teams (with a subcategory of cohesion), dialogue and communication (with subcategories of self-leadership, ability to listen and empathy), leadership of different profiles, confidence (with subcategories of efficiency), and goals (with subcategories of transmission capacity and identification with the group) (Table 5).

**TABLE 5:** Comparative categories and subcategories and illustrative quotes for current participants and alumni.

Alumni		Illustrative quotes
Categories	Subcategories	
Heterogeneity in teams		To a large extent, heterogeneity is the mother of big teams. The combined talent of the team is greater than the sum of talents. However, alone, it is not worth it.
	Cohesion	I have understood that each individual is different and that we have different needs. The way we talk and address each other should vary if we want these messages to reach a deeper level. It is not the ability we have to attract people but the ability we have to adapt to them.
Dialogue and communication		Increasing communication to realize that different types of people have different needs has led me to generate environments where new voices, ideas, and concepts arise.
	Self-leadership, ability to listen, and empathy	Among other things, I have adopted the ability to understand different types of profiles, and this has helped me to understand their personal goals reflected in their daily actions within the organization.
Leadership of different profiles, confidence		Yes, one must first gain the ability to detect people who wish to develop leadership, and who are aligned with the leader profile required by the rest of the team and the company itself, in order to allow them to lead projects and teams of people, which in turn allows them to demonstrate their strengths as leaders.
	Efficiency	People are more effective and efficient when they have a purpose.
Goals		Yes. When people participate in defining the values and strategy of the company (it is not something exclusive to management), we manage to create a climate of confidence and a shared project.
	Transmission and identification with the group	The alignment of one’s personal purpose with that of the company is fundamental. That is why a shared vision is very relevant to making team leadership successful.

**Current participants**		**Illustrative quotes**

Heterogeneity in teams		A heterogeneous group is richer in ideas and allows each person to be located where the best performance is obtained.
Dialogue and communication		Communicate in order to transmit the objectives in the best possible way and thus “convince” your team.
Goals and consensus		When an objective is based on consensus, it is more likely to succeed. Having a clear company mission and transmitting it properly to the entire organization aligns the objectives of its members and motivates them to work together, thus greatly increasing the chances of success.
Group membership		The leader must transmit passion and enthusiasm, spreading them to everyone within the organization, and exhibit an empathy that makes workers identify with the project.

Although alumni participants are present in some subcategories, the responses to the open-ended questionnaires are analyzed and divided into five main categories: heterogeneity in teams, dialogue and communication, goals and consensus, role of leadership and different profiles, and confidence in following.

### Heterogeneity in Teams

Responses by current participants indicate that they believe that heterogeneity makes teams more competitive and richer in ideas, as illustrated in the following quotation: “A homogeneous group may be easy to lead, but I think it is not valid for competitiveness. A heterogeneous group is richer in ideas and allows each person to be located in the area where the best performance is obtained.”

Meanwhile, the responses by alumni participants are more numerous. They highlight the role of heterogeneity in identifying possible challenges and enriching decision-making, the wealth generated by heterogeneity, and aspects that should be accompanied by heterogeneity, such as a common purpose and a high degree of confidence:

“To a large degree, heterogeneity is the mother of big teams. The combined talent of the team is greater than the sum of its talents. But alone, it is not worth it. It must be accompanied by a common purpose and a high degree of confidence among its members.”

Heterogeneity has been directly linked with success, not only in terms of economic success but also in relation to the wealth and enrichment of decision-making processes within the company. A well-applied diversity strategy enables the creation of well-balanced teams, which is in fact connected to efficiency. One of the participants highlighted this diversity as follows: “Success lies in diversity, since it helps one understand and address the possible challenges that exist from different angles, and that always enriches decision-making and even execution.”

“Diversity brings a lot of wealth, not only in technical capabilities but in everything else. And you have to take into account the characteristics of each one when creating the equipment.”

### Dialogue and Communication

Current participants believe that communication is crucial in order to “transmit the objectives in the best possible way and thus ‘convince’ your team.” Compared with current participants, alumni participants believe that after their EMBA, they have changed their perceptions of how people understand the objectives in an organization in the sense of “objectives and missions.” In this regard, one of the participants said, “by increasing communication, by realizing that different types of people have different needs, this has led me to generate environments where new voices, ideas, and concepts come up.”

Some participants link the importance of communication to the ability of leaders to listen and empathize. To transmit objectives within a company, it is necessary to demonstrate this empathy with all members. If you can handle the great diversity of members and manage their abilities, your work may have a greater impact. This is illustrated by one participant as follows:

“I have adopted the ability to understand the different types of profiles, and it has helped me to understand their personal goals reflected in their daily actions within the organization. This has allowed me, through team management learned in the Master’s, to build their strengths and focus them as far as possible in order to align these needs and personal preferences within the organization, thus maximizing their efficiency and motivation.”

### The Role of Leadership in Relation to Different Profiles and Confidence

The role of leadership was linked to the notions of passion, enthusiasm, and empathy by one of the current participants: “transmit passion and enthusiasm, spreading them to everyone within the organization, in addition to showing empathy that makes workers identify with the project.”

The leader’s role was highlighted as a basic pillar for the effective development of a company. The difference between a good and a bad leader is fundamental for achieving success or, conversely, failing:

“Good leadership can make a difference between the success and failure of an organization. The figure of the leader is important in making a difference, since it provides the differential point when making strategies, defining objectives, etc. Similarly, bad leadership can condemn an organization to failure.”

Alumni, after studying for their master’s, connected the role of leadership to motivating people’s participation, getting the best out of each member, and situational and shared leadership, as follows:

“I understand leadership in my company as a situational and shared leadership in which sometimes one leads a project and, on another occasion, leads another with the support and help of others (…); it is the ability to connect with your team, to motivate and get the best out of each member.”

In addition, after obtaining his master’s degree, one alumnus believes that he has been able to improve the context for different people in his organization to develop leadership. This is clearly stated as follows:

“First, obtaining the ability to detect people who wish to develop leadership, and who are aligned with the leader profile required by the rest of the team and the company itself, in order to allow them to lead projects and teams of people, which in turn allows them to demonstrate their strengths as leaders.”

Although confidence is an important element for alumni, it is not mentioned by current participants. One alumnus links the idea of confidence with the notion of identifying different qualities in a great diversity of members. In other words, you need confidence if you want to be successful within the organization. “Each person is a treasure different from the rest of the team. It is part of my job and my responsibility to identify those qualities and give them a value for the organization.”

“To manage teams, it is essential to know each of the people and whether they have enough confidence and motivation to express their thoughts, feelings, and particular perspectives on their work and the company’s global aims to manage teams and to identify each of their profiles.”

Some alumni link the notion of confidence with common goals and efficiency. One of them replied that “people are more effective and efficient when they have a purpose.” It is also important to highlight that alumni not only think about the members of their organizations but also about how they can improve their role as leaders in the company. Along these lines, one alumnus said that

“Not only has it covered the initial needs and expectations, but it has also allowed me to know myself as a leader and thus build up my strengths and turn my weaknesses into opportunities for improvement, facilitating the daily management of the teams.”

### Goals and Consensus

Current participants noted the importance of consensus to reach success within the company. Effective leadership must seek consensus in all undertakings. A current participant commented: “When an objective is based on consensus, it is more likely to succeed.”

Another current participant discussed the need to have the goals of the company clarified in order to achieve success. Having a clear company mission is fundamental. In the words of one current participant, “Having a clear company mission and transmitting it properly to the entire organization aligns the objectives of its members and motivates them to work together, greatly increasing the chances of success.”

One alumnus introduced an important element regarding the goals of a company. In this case, the participant focuses on the way in which the goals are specified within a company. If you encourage the participation of all members, independently of their status in the company, you will reach common goals and achieve a better environment within the company. “When people participate in defining the values and strategy of the company (it is not something exclusive to Management), we manage to create a climate of confidence and a shared project.”

Directly related to goals and consensus, one alumnus mentioned transmission capacity and identification with the group as two key elements. “The alignment of one’s personal purpose with that of the company is fundamental. That is why a shared vision is very relevant to making team leadership a success.”

Lastly, one alumnus noted another key element related to the importance of working as a large team within the company. To do so, one must look for common goals within the company’s great diversity and heterogeneity:

“In organizations, it is customary to promote teamwork within each of the teams, but this can also encourage the isolation of teams among themselves and the loss of a common goal. Within our organization, work among teams is encouraged through different methods toward a common vision and purpose and so that all people are able to recognize the work of others.”

## Discussion

The aim of this study was to identify and analyze the improvement in leadership skills and aptitudes before and after the implementation of an excellence EMBA program, particularly in relation to two components linked to leader behavior: goals/purposes and people/composition. Understanding these components, which are analyzed in depth throughout this study, becomes crucial to exploring the impact of leadership on organizations.

Using data analysis, we have identified that the leadership skills and aptitudes resulting from the implementation of the EMBA have had a positive impact on alumni compared with current EMBA participants. The leadership courses included in the EMBA have clear relationships with the components identified in the scientific literature, and some aspects may create synergies with a dialogic dimension of leadership addressed to achieve social impact. Such improvements in leadership skills and aptitudes indicate the potential for achieving social impact in their organizations, which is coherent with recent developments in the field of social impact research (Reale et al., 2017; European Commission, 2018; Pulido et al., 2018).

After finalizing the EMBA, Alumni highlighted heterogeneity and cohesion as key elements in advancing toward a better-performing and more competitive organization. Professional performance is clearly linked with a multidisciplinary team structure. Both heterogeneity and cohesion have been addressed in the leadership literature, highlighting diversity as a key pillar of reaching optimum performance (Hoch, 2014) and linking educational diversity to effective team performance (Kearney and Gebert, 2009).

Dialogue and communication have been linked by participants to self-leadership, the ability to listen, and to empathy (Berkovich, 2014). This is covered in more detail and in more numerous contributions by alumni than by current EMBA participants. The scientific literature explains that this occurs because the capacity for dialogue maximizes efficiency and motivation in organizations (Chen and Kanfer, 2006). This explains why the EMBA has helped alumni maximize their work in their respective companies through team management, with dialogue and communication as their two key pillars, in line with dialogic leadership (Padrós and Flecha, 2014). Furthermore, according to the literature, dialogue and communication may influence the promotion of creativity linked to leadership (Cai et al., 2018) because it motivates team members to develop company objectives, thus favoring potential social impact. Along with this aspect, it is also important to mention that motivation has been highlighted by psychological research and studies on leadership in organizations. Chen (2007) corroborated the need for leadership to motivate members, thus facilitating the emergence of creative proposals directly related to higher performance and impact.

Effective leadership has been linked with the notion of a good manager, thus maximizing the efficiency and performance in the company. To move in this direction, one must identify the company’s main goals and base actions on consensus, sharing the company’s mission and purpose. That mission must be transmitted properly to all members and, to do so, an effective leadership approach is crucial, one that motivates all members and increases an organization’s potential social impact.

## Limitations and Future Research

This is an exploratory study of the improvement in leadership skills and aptitudes in an excellence EMBA program, comparing insights from alumni who completed the program and current EMBA participants. The study is limited by the wide period covered, as it includes alumni from the 10 completed offerings of the EMBA, although most respondents belong to the most recent offering. Another limitation is that no previous studies have been carried out on this specific EMBA concerning leadership, which made it difficult for us to create synergies with similar studies.

Despite these limitations, the study illustrates the improvement in the leadership skills and aptitudes of alumni, the synergies with dialogic leadership, and the potential social impact in their organizations. Future research analyzing the social impact achieved could not only provide evidence of advancements toward Goal 8 of the Sustainable Development Goals but also provide ways of improving the excellence of the EMBA.

## Conclusion

The results demonstrate an improvement in leadership skills and aptitudes as a result of the development of an excellence EMBA program, which contributes to the potential social impact of organizations. There are several areas in particular in which alumni have increased their effective leadership compared with current EMBA participants: heterogeneity in teams (with subcategories of cohesion), dialogue and communication (with subcategories of self-leadership, ability to listen, and empathy), leadership of different profiles, confidence (with subcategories of efficiency), and goals (with subcategories of transmission capacity and identification with the group). These features, linked to more effective leadership, arise as a result of having completed the EMBA program. Our findings contribute to improving the EMBA itself, as our results will be shared with the academic staff of the program responsible for the content concerning leadership.

## Data Availability Statement

All datasets generated for this study are included in the article/supplementary material.

## Ethics Statement

Alumni were invited to participate in order to obtain a better understanding of their professional development after completing the EMBA and for EMBA accreditation. Current participants of the EMBA were invited, by email and personally, to participate in the context of EMBA accreditation. In both cases, participants were informed about the potential publication of the results in order to enhance the prestige of the EMBA. All participants were informed that their participation was anonymous and voluntary and that data would be treated with confidentiality and used solely for research purposes. Ethical requirements were addressed following the Ethics Review Procedure established by the European Commission (2013) for EU research, the Data Protection Directive 95/46/EC, and the Charter of Fundamental Rights of the European Union (2000/C 364/01). The above procedure was reviewed and approved by the Ethics Committee of the Community of Research on Excellence for All.

## Author Contributions

JC was responsible for data collection and contributed to the discussion and conclusions. AA conceptualized and designed the article and revised and approved the manuscript. MJ and MG collaborated in data analysis and in elaborating the article.

## Conflict of Interest

The authors declare that the research was conducted in the absence of any commercial or financial relationships that could be construed as a potential conflict of interest.
